# Virey’s Objections to Liebig’s Theory of the Uses of Respiration and of the Food

**Published:** 1843-01

**Authors:** 


*Virey's Objections to Liebig's Theory of the Uses of Respiration and of the Food.*—Liebig maintains that the chief use of the food is to supply carbon and hydrogen, which, uniting with the oxygen absorbed from the air, give rise to the generation of animal heat. He consequently holds that there is a certain fixed relation between the amount of food consumed, and the quantity of carbon and hydrogen thrown off at the lungs. Mr. Virey opposes this theory, as contrary to common observations, as, even though it be allowed to be applicable to mammalia, birds, and reptiles, it is by no means to those animals which respire by means of branchiæ. Thus all animals with branchiæ consume but little oxygen, comparatively speaking, and yet many of them devour very great quantities of food. Even the largest and most voracious of the reptiles, as the alligators, crocodiles, &c., which devour enormous quantities of food, under a burning climate too, respire feebly with their vesicular lungs, and consume but little oxygen.

Fishes, whose blood is but imperfectly oxygenated by the branchial apparatus, are perhaps among the most voracious of animals, and yet, according to Liebig's theory, they ought to eat little, because they consume little oxygen.

The same holds true of the Mollusca. The cuttle-fish, *buccinum, strombus, murex,* &c., grow to a large size; but their respiration is very imperfect, and yet they are great flesh-eaters. The Crustacea, again, as the crabs, lobsters, &c., grow rapidly, because they are great eaters; but their branchial apparatus is not fitted to consume much oxygen.

In all these animals assimilation takes place very rapidly, notwithstanding their feeble respiratory powers; and they are, besides, by no means deficient in activity or muscular powers, though their flesh be but feebly azotized or animalized, and their blood is always cold.

If it be one of the characters of vitality, that the more perfect this principle is, the greater is the number of germs, or eggs, or fœtuses produced, then, quite contrary to Liebig's theory, the number of germs produced is in the inverse ratio of the perfection of the respiratory functions. Fishes and mollusca deposit their spawn or eggs by millions; but the mammalia, and even the birds, whose respiratory functions are the most perfect, are in this respect infinitely behind these. On the other hand, it is seen that the number of germs or eggs is rather proportioned to the nutrition received; for the amount of food taken is not proportioned to the respiration in the animal kingdom.

Virey therefore concludes, that the vital force or central nervous energy has more to do with the production of animal heat than the consumption of carbon at the lungs, and this for three special reasons;—1st, Because a fecundated egg resists a freezing temperature longer than one which has not been fecundated. 2d, That a hybernating insect, reptile, or animal, or even trees during winter, by the sole influence of a vital power, resist a freezing temperature, whereas the same animals, if dead, would be instantly frozen. 3d, That many mammalia and birds keep themselves warm even in the most rigorous winters under the Pole, not in consequence of a greater amount of oxygen consumed, nor by a greater amount of muscular activity, but in consequence of a more abundant highly azotized or animalized nourishment.—*Jour. de Pharm., May*, 1842.

